# Collecting e‐cigarette aerosols for in vitro applications: A survey of the biomedical literature and opportunities to increase the value of submerged cell culture‐based assessments

**DOI:** 10.1002/jat.4064

**Published:** 2020-10-04

**Authors:** Daniel J. Smart, Gary Phillips

**Affiliations:** ^1^ PMI R&D, Philip Morris Products SA Neuchâtel Switzerland; ^2^ Imperial Brands PLC Bristol UK

**Keywords:** aerosol collection, e‐cigarette, in vitro assays, literature survey, submerged cell cultures

## Abstract

Electronic nicotine delivery systems (ENDS) are being developed as potentially reduced‐risk alternatives to the continued use of combustible tobacco products. Because of the widespread uptake of ENDS—in particular, e‐cigarettes—the biological effects, including the toxic potential, of their aerosols are under investigation. Preclinically, collection of such aerosols is a prerequisite for testing in submerged cell culture‐based in vitro assays; however, despite the growth in this research area, there is no apparent standardized collection method for this application. To this end, through an Institute for in vitro Sciences, Inc. workshop initiative, we surveyed the biomedical literature catalogued in PubMed® to map the types of methods hitherto used and reported publicly. From the 47 relevant publications retrieved, we identified seven distinct collection methods. Bubble‐through (with aqueous solvents) and Cambridge filter pad (CFP) (with polar solvents) collection were the most frequently cited methods (57% and 18%, respectively), while the five others (CFP + bubble‐through; condensation; cotton filters; settle‐upon; settle‐upon + dry) were cited less often (2–10%). Critically, the collected aerosol fractions were generally found to be only minimally characterized chemically, if at all. Furthermore, there was large heterogeneity among other experimental parameters (e.g., vaping regimen). Consequently, we recommend that more comprehensive research be conducted to identify the method(s) that produce the fraction(s) most representative of the native aerosol. We also endorse standardization of the aerosol generation process. These should be regarded as opportunities for increasing the value of in vitro assessments in relation to predicting effects on human health.

## Abstract 2

Collection of e‐cigarette aerosols is a prerequisite to enable testing in submerged culture‐based in vitro assays; however, there is no standardized method for this. Thus, we surveyed the biomedical literature to map the types of published methods. Bubble‐through and Cambridge filter pad methods were most common, although there was heterogeneity among other parameters, and moreover, the resulting fractions were only minimally characterized. Comprehensive research is required to identify the method(s) that produce the fraction(s) most representative of the native aerosol.

## 1 INTRODUCTION

Electronic nicotine delivery systems (ENDS) are being developed as potentially reduced‐risk alternatives to the continued use of combustible tobacco products (Brandon et al., 2015; Farsalinos & Polosa, 2014). Electronic cigarettes (e‐cigarettes)—one of the most widely known ENDS—consist of a battery‐powered device that heats an “e‐liquid” contained inside an atomizer, which leads to generation of an inhalable aerosol upon puffing by the user (McRobbie, Bullen, Hartmann‐Boyce, & Hajek, 2014). E‐cigarette devices can vary extensively in terms of design and functionality (Breland et al., 2017). Similarly, e‐liquids are also highly diverse and can contain different levels of nicotine, flavoring agents, and humectants such as propylene glycol and vegetable glycerin (Brown & Cheng, 2014).

The recent rise in the use of e‐cigarettes around the world has brought the toxicity of their aerosols into focus (Callahan‐Lyon, 2014; Orr, 2014). Many institutes, including those from industry, academia, and government, are conducting research to understand their toxicological hazard and risk potential. As in other areas, much of the preclinical research on e‐cigarette‐derived aerosols is performed in in vitro cell culture models because they are relatively inexpensive (compared with animals), amenable to different types of higher‐throughput analyses, supportive of the 3Rs principles (to Replace, Reduce, and Refine animal usage in scientific experiments), and importantly, the data generated from these models are potentially translatable to higher levels of biological organization. Significantly, in vitro toxicology data can influence the development of an e‐cigarette device or e‐liquid formulation. However, because many in vitro assays are conducted in submerged two‐dimensional cell cultures, the aerosol generated from an e‐cigarette must first be collected before it can be applied to the cell model under investigation. This challenge was originally faced by researchers seeking to investigate the toxicity of cigarette‐derived smoke in vitro (Bradford, Harlan, & Hanmer, 1936). Ultimately, relatively standardized processes were developed whereby the smoke from combustible tobacco products was generated via smoking machines and subsequently collected in several ways (reviewed in Klus, Boenke‐Nimphius, & Müller, 2016), including (a) total particulate matter or condensate captured on a Cambridge (glass fiber) filter pad (CFP) and desorbed with dimethyl sulfoxide (DMSO); (b) condensate captured via electrostatic precipitation (EP) and solubilized in DMSO; (c) condensate captured in a cold trap; and (d) aqueous solution (AQ)‐soluble gas–vapor phase (GVP) constituents captured in phosphate‐buffered saline (PBS). Some of these collection methods can be applied in tandem—for example, sequential CFP‐ or EP‐ and AQ‐mediated trapping—and they can produce fractions that are broadly representative of the composition of tobacco smoke when considered as a whole (Klus, Boenke‐Nimphius, & Müller, 2016). Although parallels can be drawn between cigarettes and e‐cigarettes in the context of smoke and aerosol collection for in vitro applications, the latter products are more contemporary than the former and, consequently, have not been subjected to the same degree of experimentation. Thus, the general level of knowledge that has been built over the decades in relation to smoke generation and collection, at present, exists only minimally for e‐cigarette‐derived aerosols. Hence, there might be scope to ameliorate various aspects of the procedures linked to e‐cigarettes.

The Institute for in vitro Sciences, Inc. (IIVS) is currently hosting a series of workshops that provide a forum for stakeholders to identify, discuss, and develop recommendations for optimal generation of test samples and use of genetic toxicology in vitro assays to support tobacco product regulatory requirements (Moore et al., 2020). This workshop series follows two previous IIVS workshops that focused on in vitro models for chronic obstructive pulmonary disease and in vitro exposure systems and related dosimetry (Behrsing et al., 2016; Behrsing et al., 2017). Because evaluation of ENDS represents a new challenge for in vitro testing, practical issues associated with these products are a major focus of the current IIVS workshop series. During the initial workshop, the participants agreed on the need for reviewing the state of the science in relation to e‐cigarette aerosol collection for in vitro applications, and, predicated on this consensus view, a survey of the biomedical literature was conducted in order to map the types of methods employed for this purpose. The present publication provides a summary of this survey. It should be noted that in vitro systems composed of cells cultured at the air–liquid interface coupled with whole aerosol exposure technologies were out of scope for this survey because it is a highly specialized area of research and merits its own dedicated review. Importantly, the opportunities arising from this survey should be exploited to improve the understanding and study of e‐cigarette‐derived aerosols in submerged cell culture‐based in vitro assays.

## 2 METHODOLOGY

### 2.1 Literature search

We conducted a search via PubMed® (https://www.ncbi.nlm.nih.gov/pubmed/)—the freely accessible literature repository containing >30 million publications from the fields of biomedicine and health (PubMed, 2020)—to identify all potentially relevant publications for subsequent evaluation. The most recent search was conducted during December 2019. We used the following search terms: ((“electronic cigarette”[All Fields] OR “electronic cigarettes”[All Fields]) OR “e‐cigarette”[All Fields]) OR (((“electronic nicotine delivery systems”[MeSH Terms] OR (((“electronic”[All Fields] AND “nicotine”[All Fields]) AND “delivery”[All Fields]) AND “systems”[All Fields])) OR “electronic nicotine delivery systems”[All Fields]) OR “e cigarettes”[All Fields]). Results were filtered by year (2013–2019: the time period when the vast majority of these publications was published) and reviews were excluded. Publications were further triaged by evaluating their abstracts (exported from PubMed®) for the following keywords: Aerosol; Capture; Collection; Condensate; Emissions; Immobiliz(s)ation; In vitro; Oxidative; Toxicity; Toxicology; Trapping; Vapo(u)r.

### 2.2 Data extraction, compilation, and visualization

Triaged publications were critically evaluated for the presence of empirical information related to collection of e‐cigarette aerosols for evaluation in in vitro assays. Relevant data were subsequently extracted and used to compile a database; the categories of data extracted are described in Tables 1 and 2. Note that, in publications that assessed more than one “item,” the items were grouped together for entry into the database. For instance, a publication that evaluated 39 e‐liquids and 5 e‐cigarette devices via two different vaping regimens would be represented in the relevant fields of the database as “39 Types,” “5 Types,” and “2 Types,” respectively. In addition, database fields were completed as “not available” (N/A), where relevant information was lacking or not explicit. Data were visualized by using Spotfire® Desktop (v7.13.0, TIBCO®, Palo Alto, CA, USA).

**TABLE 1:** Selected database statistics

Parameter	Details
Publications	47
Individual collection methods	49
Primary institutes	≥34
Publication year range	2013–2019
Research areas	Immunology; inflammation; oral health; oxidative stress; tissue repair; toxicology; vascular

**TABLE 2:** Summary of the collection method‐related information from the 47 publications and additional data

Collection method	Manuscript reference	PMID	Collection solvent(s)	Fraction(s)	E‐cigarette device	E‐liquid	Smoking machine	Puffs	Vaping regimen	Nicotine quantified
Bubble‐through	Breheny, Oke, Pant, & Gaça, 2017	28 444 993	Medium	AQE	Vype ePen	Blended Tobacco	SM‐450	10	CRM No. 81	Yes
	Rayner, Makena, Prasad, & Cormet‐Boyaka, 2019	31 166 129	Medium	AQE	Innokin VV4/Nautilus tank	Tobacco row	N/A	N/A	55‐mL vol, 5‐s draw, 30‐s interval	Yes
	Munakata et al., 2018	30 227 175	Medium	AQE	2 types	N/A	N/A	300	HCI	Yes
	Taylor et al., 2017	28 658 606	Medium	AQE	2 types	Blended tobacco	RM20H	10	CRM No. 81	Yes
	Bengalli, Ferri, Labra, & Mantecca, 2017	29 053 606	Medium	AQE	Kit iSimple Ribilio/C14 Passthrough	12 types	TRUST‐iCERT	200	55‐mL vol, 3‐s draw, 60‐s interval	No
	Behar et al., 2016	27 633 763	Medium	AQE	2 types	39 types	N/A	24	2 types	No
	Farsalinos et al., 2013	24 135 821	Medium	AQE	2 types	21 types	Vacuum	N/A	2 types	No
	Ganapathy et al., 2017	28 542 301	HEPES‐buffered saline	AQE	2 types	5 types	Vacuum	N/A	HCI	Yes
	Rubenstein, Hom, Ghebrehiwet, & Yin, 2015	26 072 673	HEPES‐buffered saline	AQE	2 types	3 types	Vacuum	N/A	N/A	No
	Ji et al., 2016	28 033 425	Medium	AQE	N/A	4 types	Homemade	N/A	33‐ to 83‐mL vol, 2‐ to 5‐s duration	No
	Anderson, Majeste, Hanus, & Wang, 2016	27 613 717	Medium	AQE	4 types	Tobacco	Vacuum	N/A	55‐mL vol, 2‐s duration, 30‐s interval	No
	Teasdale, Newby, Timpson, Munafò, & White, 2016	27 137 404	Medium	AQE	Aerotank Mini/iStick battery	Haven fluid USA Mix	N/A	5	5.8‐mL vol, 5‐s draw, 10‐s interval	Yes
	Taylor et al., 2016	27 690 198	Medium	AQE	2 types	Blended tobacco	RM20H	10	CRM No. 81	Yes
	Omaiye, McWhirter, Luo, Pankow, & Talbot, 2019	30 896 936	Medium	AQE	JUUL EC	8 types	Peristaltic pump	N/A	43‐ to 56‐mL vol, 4.3‐s draw, 60‐s interval	Yes
	Leslie et al., 2017	28 470 141	Medium	AQE	5 types	15 types	Diaphragm pump	14	ISO	No
	Rankin et al., 2019	30 957 912	Medium	AQE	Joyetech eVic VT/eGo ONE Mega atomizer	2 types	Water aspirator	13	1.5‐s draw, 30‐s interval	No
	Kaisar, Sivandzade, Bhalerao, & Cucullo, 2018	29 879 439	PBS	AQE	N/A	N/A	SCSM	8	FTC	No
	Bharadwaj, Mitchell, Qureshi, & Niazi, 2017	27 875 752	Medium	AQE	N/A	Classic tobacco	Vacuum	N/A	N/A	Yes
	Yu et al., 2015	26 547 127	Medium	AQE	2 types	4 types	N/A	N/A	N/A	No
	Di Biase, Attorri, Di Benedetto, & Sanchez, 2018	30 575 566	FBS	AQE	Kelvin	2 types	Vacuum	N/A	10‐s draw	No
	Higham et al., 2016	27 184 092	Medium	AQE	3 types	6 types	Peristaltic pump	N/A	2 types	No
	Higham, Bostock, Booth, Dungwa, & Singh, 2018	29 615 835	Medium	AQE	V5/CE5 clearomiser/VIP battery	USA tobacco	Peristaltic pump	N/A	N/A	No
	Hom et al., 2016	27 096 416	HEPES‐buffered saline	AQE	2 types	5 types	Vacuum	N/A	N/A	No
	Otręba, Kośmider, Knysak, Warncke, & Sobczak, 2018	29 665 082	Medium	AQE	eGo‐3 twist battery/bottom headed clearomizer	6 types	Palaczbot	30	70‐mL vol, 1.8‐s draw, 17‐s interval	No
	Raez‐Villanueva, Ma, Kleiboer, & Holloway, 2018	30 048 688	Medium	AQE	EVOD Kanger‐Tech	2 types	N/A	N/A	N/A	Yes
	Ween, Whittall, Hamon, Reynolds, & Hodge, 2017	28 867 672	Medium	AQE	EVOD‐2	10 types	N/A	50	3‐s draw, 5‐s interval	No
	Zahedi et al., 2019	31 200 115	Medium	AQE	N/A	2 types	UoK ASM	N/A	4.3‐s draw, 60‐s interval	No
	Zhao et al., 2018	29 102 637	Medium	AQE	N/A	2 types	ECAG	N/A	2 types	Yes
CFP	Breheny, Oke, Pant, & Gaça, 2017	28 444 993	DMSO	DMSO‐sol ACM	Vype ePen	Blended tobacco	LM20X	40	CRM No. 81	Yes
	Rayner, Makena, Prasad, & Cormet‐Boyaka, 2019	31 166 129	Medium	AQ‐sol ACM	Innokin VV4/Nautilus tank	Tobacco row	N/A	N/A	55‐mL vol, 5‐s draw, 30‐s interval	Yes
	Thorne et al., 2016	27 908 385	DMSO	DMSO‐sol ACM	Vype ePen	Blended tobacco	LM20X	N/A	CRM No. 81	No
	Misra, Leverette, Cooper, Bennett, & Brown, 2014	25 361 047	PBS	AQ‐sol ACM	blu eCigs	4 types	Vitrocell VC10	N/A	HCI	Yes
	Husari et al., 2016	26 272 212	Medium	AQ‐sol ACM	V4L CoolCart/Vapor Titan Soft Touch battery	Strawberry	ONARES	N/A	80‐mL vol, 4‐s duration, 14‐s interval	No
	Shaito et al., 2017	29 079 789	Medium	AQ‐sol ACM	V4L CoolCart	Strawberry	ONARES	N/A	80‐mL vol, 4‐s duration, 14‐s interval	No
	Dalrymple et al., 2018	30 346 667	DMSO	DMSO‐sol ACM	NVP	Twilight tobacco	LM20X	200	CRM No. 81	No
	Ito et al., 2019	31 400 404	DMSO	DMSO‐sol ACM	Vype ePen	Blended tobacco	RM20D	N/A	CRM No. 81	Yes
	Thorne et al., 2019	31 163 219	DMSO	DMSO‐sol ACM	N/A	N/A	RM200a	60	CRM No. 81	Yes
CFP + bubble‐through	Takahashi et al., 2018	29 158 044	DMSO + PBS	DMSO‐sol ACM + AQ‐sol GVP	NTV	N/A	Rotary	70	HCI	No
Condensation	Scott et al., 2018	30 104 262	N/A	Conden‐sate	Kanger	2 types	N/A	N/A	3‐s draw, 30‐s interval	Yes
	Clapp et al., 2017	28 495 856	N/A	Conden‐sate	LAVABOX DNA 200/SMOK TFV4/TF‐CLP2 Clapton coil	7 types	Peristaltic pump	20	30‐s Interval	No
	Lei, Lerner, Sundar, & Rahman, 2017	28 256 533	N/A	Conden‐sate	eGO/eGo Vision Spinner battery	4 types	Peristaltic pump	N/A	4‐s draw, 30‐s interval	No
	Schweitzer et al., 2015	25 979 079	N/A	Conden‐sate	Innokin iClear 16	3 types	Vacuum	N/A	N/A	No
	Sun, Kosinska, & Guttenplan, 2019	31 373 329	25% DMSO/Water/PBS	AQ‐sol condensate	2 types	2 types	ASPECG pump	N/A	ISO	Yes
Cotton filters	Miyashita et al., 2018	29 437 942	PBS	AQ‐sol ACM	RBC CE5 Clearo‐mizer	2 types	Peristaltic pump	25	N/A	No
Settle‐upon	Shivalingappa, Hole, Westphal, & Vij, 2016	26 377 848	Medium	AQE	Kanger EVOD	Flavorless	Motor	N/A	N/A	No
	Behar, Wang, & Talbot, 2018	28 596 276	Medium	AQE	iClear16D dual coil/Innokin iTaste MVP 3.0 battery	45 types	Peristaltic pump	N/A	56‐mL vol, 4.3‐s draw, 60‐s interval	No
	Alanazi, Park, Chakir, Semlali, & Rouabhia, 2018	29 800 583	Medium	AQE	EMOW	Smooth Canadian tobacco	Peristaltic pump	N/A	10‐s draw, 30‐s interval	No
	Zahedi, Phandthong, Chaili, Remark, & Talbot, 2018	30 032 837	Medium	AQE	N/A	2 types	N/A	N/A	4.3‐s draw, 60‐s interval	No
Settle‐upon + dry	Tommasi, Bates, Behar, Talbot, & Besaratinia, 2017	29 191 599	MtOH/DMSO	MtOH‐/DMSO‐sol extract	3 types	N/A	Peristaltic pump	10	3 types	No

Abbreviations: ACM, aerosol collected matter; ASPECG, aerosol single port electronic cigarette generator; AQ, aqueous solution; AQE, aqueous extract; CRM No. 81, CORESTA recommended method number 81 (square wave puff profile, 55‐mL vol, 2‐s draw, 28‐s interval); DMSO, dimethylsulfoxide; ECAG, e‐cigarette aerosol generator; FTC, Federal Trade Commission (bell‐shaped puff profile, 35‐mL vol, 2‐s draw, 58‐s interval); FBS, fetal bovine serum; GVP, gas–vapor phase; HCI, Health Canada Intensive (bell‐shaped puff profile, 55‐mL vol, 2‐s draw, 28‐s interval); HEPES, 4‐(2‐hydroxyethyl)‐1‐piperazineethanesulfonic acid; ISO, International Organization for Standardization 3308, (bell‐shaped puff profile, 35‐mL vol, 2‐s draw, 58‐s interval); MtOH, methanol; N/A, not available; NTV, novel tobacco vapor product; NVP, novel vapor product; ONARES, oro‐nasal respiratory exposure system; PBS, phosphate‐buffered saline; SCSM, single cigarette smoking machine; s, seconds; sol, soluble; vol, volume; UoK ASM, University of Kentucky analytical smoking machine.

## 3 RESULTS

### 3.1 General database statistics

The initial search retrieved 4561 publications. From these, further keyword triaging identified 1543 publications. Upon inspection, 47 publications from the 1543 were found to contain relevant empirical data, while the remaining were rejected because of lack of direct relevance to e‐cigarette aerosol collection for in vitro application and/or absence of empirical information. Interestingly, two of these publications reported two distinct collection methods each (Breheny et al., 2017; Rayner et al., 2019). Thus, in total, there were 49 individual collection methods itemized in the database. Selected database statistics are described in Table 1.

### 3.2 Collection method‐related information

**FIGURE 1:**
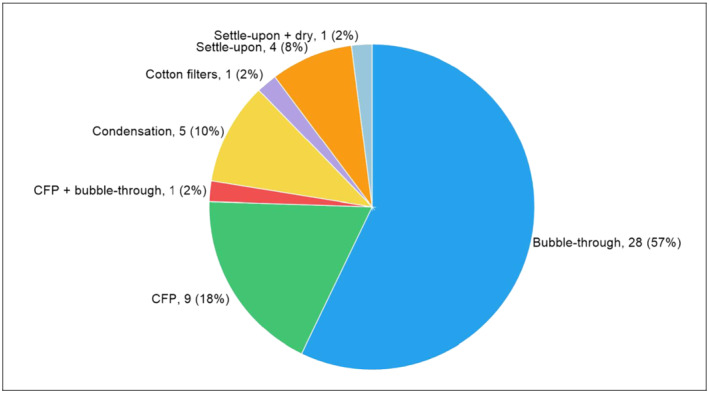
Types of methods employed for collection of e‐cigarette‐derived aerosols for in vitro research. CFP, Cambridge filter pad

Table 2 provides a summary of the relevant data. Seven distinct collection methods were reported in the 47 publications; these were defined as “bubble‐through,” “CFP,” “CFP + bubble‐through,” “condensation,” “cotton filters,” “settle‐upon,” and “settle‐upon + dry.” Each collection method is described in the Section 4, while graphical illustrations are presented in another publication emanating from the IIVS workshop series (Wieczorek et al., 2020). Bubble‐through and CFP were the most frequently cited collection methods (57% and 18%, respectively), while the others were cited less often (2–10%) (Figure 1).

**FIGURE 2:**
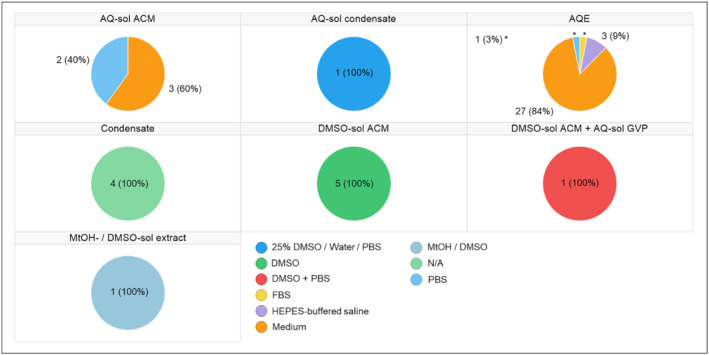
Solvents used in collection of e‐cigarette aerosols and, consequently, the fraction(s) evaluated in in vitro research. AQ, aqueous solution; AQE, aqueous extract; ACM, aerosol collected matter; DMSO, dimethylsulfoxide; FBS, fetal bovine serum; GVP, gas–vapor phase; HEPES, 4‐(2‐hydroxyethyl)‐1‐piperazineethanesulfonic acid; MtOH, methanol; N/A, not available; PBS, phosphate‐buffered saline; sol, soluble

In addition, eight different solvent systems were used in these collection methods, including “25% DMSO/Water/PBS,” “DMSO,” “DMSO + PBS,” “fetal bovine serum (FBS),” (4‐(2‐hydroxyethyl)‐1‐piperazineethanesulfonic acid) (HEPES)‐buffered saline,” “medium,” “methanol (MtOH)/DMSO,” and “PBS” (Table 2). Note that four studies applied the “condensation” collection method without a solvent and, thus, were represented with N/A in these fields of the database. Consequently, on the basis of the collection methods and solvents employed in the 47 publications, we defined seven different categories of aerosol fraction(s): “AQ‐soluble aerosol collected matter (ACM),” “AQ‐soluble condensate,” “aqueous extract (AQE),” “condensate,” “DMSO‐soluble ACM,” “DMSO‐soluble ACM + AQ‐soluble GVP,” and “MtOH‐/DMSO‐soluble extract” (Table 2). A graphical summary of this information is provided in Figure 2.

### 3.3 Additional findings

**FIGURE 3:**
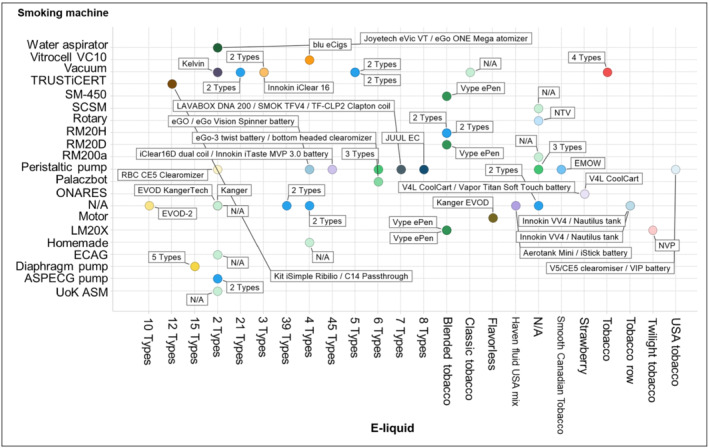
E‐liquids, smoking machines, and e‐cigarette devices (annotated within the figure) used in generation of aerosol(s) for in vitro research. ASPECG, aerosol single‐port electronic cigarette generator; N/A, not available; NTV, novel tobacco vapor product; NVP, novel vapor product; ONARES, oro‐nasal respiratory exposure system; SCSM, single cigarette smoking machine; UoK ASM, University of Kentucky analytical smoking machine

**FIGURE 4:**
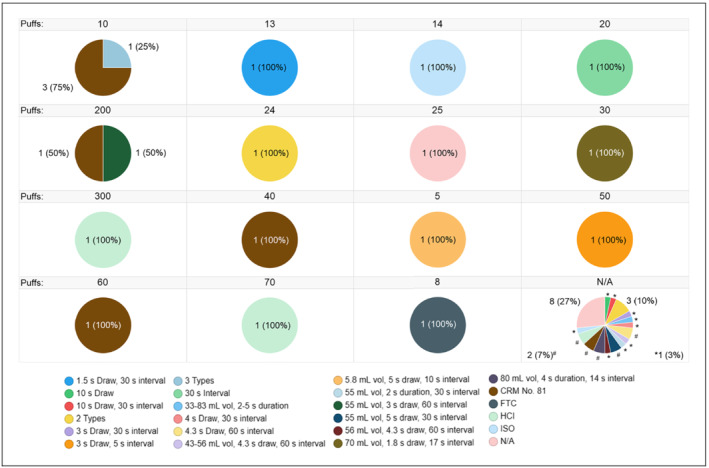
Number of puffs and vaping regimens used in the generation of aerosol(s) that were collected for in vitro research. CRM No. 81, CORESTA recommended method number 81; FTC, Federal Trade Commission; HCI, Health Canada Intensive; ISO, International Organization for Standardization 3308; N/A, not available; s, seconds; vol, volume

In general, there was large heterogeneity among the other e‐cigarette aerosol generation‐related parameters in the 47 publications. For example, the collected aerosols were generated from a multitude of e‐cigarette devices and e‐liquids via numerous different commercially available smoking machines or laboratory‐built apparatuses (Figure 3). Furthermore, there was also diversity in the vaping regimens and number of puffs applied for aerosol generation (Figure 4). In addition, a number of studies performed very limited chemical characterization of the collected e‐cigarette aerosols (i.e., nicotine quantification) (Table 2).

## 4 DISCUSSION

Like in other areas of applied research, in vitro data have an important role to play in helping us comprehend the toxic potential of e‐cigarette‐derived aerosols in humans, while also supporting the 3Rs principles of scientific animal experimentation. Such data can help not only more readily identify hazards in an animal‐cognizant manner but also potentially delineate modes‐of‐action (Ramirez et al., 2018; Shukla, Huang, Austin, & Xia, 2010); ultimately, this information will add to the weight‐of‐evidence that informs the risk assessment of e‐cigarettes in relation to human health. Although sophisticated approaches involving three‐dimensional organotypic respiratory tract cell cultures and whole aerosol exposure systems that partially recapitulate physiologically relevant exposure in humans might eventually become the key model for studying aerosol‐associated toxicity, they are, at present, in their infancy and still require further exploration and validation (Bishop et al., 2019; Czekala et al., 2019; Iskandar et al., 2019; Mathis et al., 2013). Ostensibly until then, submerged two‐dimensional cell culture‐based assays will represent the core of in vitro assessments, in particular, for the internationally accepted tests that are used for identifying genotoxic and cytotoxic hazards, such the in vitro micronucleus, mouse lymphoma, bacterial mutagenicity, and neutral red uptake assays (INVITTOX, 1990; OECD, 1997; OECD, 2016a, 2016b). However, for the in vitro data to hold appreciable value in this context, it is vital that the captured aerosol is representative of its native aerosol. Significantly, underpinning this requirement is the collection method and solvent(s) employed. To this end, we surveyed the biomedical literature from the PubMed® repository for the approaches used by different laboratories across the world in their published in vitro research on collected e‐cigarette‐derived aerosols.

Among the 47 relevant publications identified in the survey (including studies Breheny et al., 2017 and Rayner, Makena, Prasad, & Cormet‐Boyaka, 2019 that described two methods each), 57% (28/49) of the collection methods were defined as “bubble‐through.” This is a method whereby aerosol generated from an e‐cigarette is bubbled into a solvent, resulting in a solution containing the aerosol constituents, which can be subsequently applied to cell cultures. Furthermore, all 28 examples found in this survey employed water‐based protic solvents—cell culture medium, FBS, PBS, or HEPES‐buffered saline—and, thus, generated AQEs. It is, therefore, hypothesized that water‐soluble constituents are captured predominately via this collection method, while other constituents—poorly and nonwater‐soluble compounds for example—are probably not. Crucially, it should be noted that there is a disturbing lack of chemical characterization data on the collected aerosols among the studies in general; this is a critical finding, which we will address later in the manuscript. Nevertheless, there is empirical support for the theory of water‐soluble constituent trapping via bubble‐through/aqueous solvent‐related methods from analytical studies on cigarette‐derived GVP collected in PBS. These reports indicate that chemicals such as carbonyls, including acids, esters, amides, imides, aldehydes, and ketones, as well as lactones, alcohols, pyridine derivatives, imidazoles, lactams, and nitrogen heterocyclic compounds can be collected effectively by the bubble‐through method (Noya et al., 2013; Schumacher, Green, Best, & Newell, 1977). In contrast, it is also recognized that numerous harmful and potentially harmful constituents from cigarette smoke (e.g., benzo[*a*]pyrene, dibenzo[*a*,*h*]pyrene, and 5‐methylchrysene) are highly lipophilic (i.e., possessing octanol–water partition coefficients [log P] > 5) (Smith & Hansch, 2000). Thus, if these types of molecules are present in the aerosols produced from e‐cigarettes, they are most likely not captured by aqueous solvent‐centric methods because of their inherent chemical properties.

The next most frequently employed collection method was CFP (18%; 9/49). In this method, e‐cigarette‐derived aerosol is pulled through a CFP to capture its constituents on the filter pad (i.e., ACM). These constituents are subsequently desorbed and solubilized in a solvent. These nine publications employed different polar solvents, both protic (cell culture medium and PBS) and aprotic (DMSO) in nature, which yielded fractions that were defined as AQ‐ or DMSO‐soluble ACM. Importantly, total particulate matter fractionated from cigarette smoke has been extensively characterized owing to the virtues of CFP‐mediated collection (Chepiga et al., 2000; Roemer et al., 2004). Thus, while the same kind of particulate matter (carbon‐based) is not present in the aerosol of e‐cigarettes because of the absence of combustion (Lampos et al., 2019), one might expect that the approach has the potential to capture a similar profile of chemicals, although the adherence capacity of aerosol components towards the CFP as well as their solubility in the applied solvent will obviously dictate which constituents finally comprise the fraction. Predicated upon published examples, this approach can potentially trap chemicals such as nicotine, glycerol, aromatic amines, and polycyclic aromatic hydrocarbons (Chepiga et al., 2000; Roemer et al., 2004). Interestingly, a recent publication reported that a CFP method is more effective in collecting a targeted set of flavor chemicals than its bubble‐through counterpart, indicating that this sorbent‐based technique might have advantages over others (Eddingsaas et al., 2018). However, one possible limitation of this capture method is that aerosol constituents not retained by the CFP are, presumably, poorly collected.

The tandem combination of CFP and bubble‐through methodologies (defined as “CFP + bubble‐through”) was cited once in this selection of curated literature (2%; 1/49). DMSO was used to solubilize and elute ACM from the CFP, while PBS was used to capture a portion of the constituents passing through the CFP; thus, it is anticipated that the two fractions contained polar/nonpolar and non‐CFP immobilized water‐soluble constituents, respectively. When aerosol is collected in this manner, toxicological assessment of both fractions—as was done in previous in vitro assessments of cigarettes and heated tobacco products (Gonzalez‐Suarez et al., 2016; Rickert, Trivedi, Momin, Wright, & Lauterbach, 2007; Roemer et al., 2015; Schaller et al., 2016)—might provide a better understanding of the hazard potential of the aerosol in its entirety.

The “condensation” collection method was cited five times (10%; 5/49), and, in four of the five studies, fractions defined as condensates were produced and then assessed in vitro. While this approach has potential advantages (e.g., circumventing the need for a collection sorbent or solvent), it is not clear which, and at what proportion, aerosol constituents (other than nicotine) are condensed and, therefore, present in the final fraction. In the fifth study, the condensate was subsequently solubilized in a solvent system comprising 25% DMSO, water, and PBS (Sun, Kosinska, & Guttenplan, 2019). However, similar to its related fractions, the composition of this particular fraction (AQ‐soluble condensate) is also not apparent.

In addition, three other aerosol collection methods were described among the selected publications. The method defined as “settle‐upon” was cited four times (8%; 4/49). Essentially, in this method, e‐cigarette aerosol is allowed to settle upon the solvent in question (cell culture medium in these specific cases), and, presumably, the aerosol constituents are eventually taken up by and solubilized in it. A similar collection method, namely, “settle‐upon + dry,” was applied once (2%; 1/49). Here, following aerosol settling and solubilization, the original solvent (MtOH) is removed by drying, and the resultant residue is resolubilized in a second solvent (DMSO). The efficiency of these methods in collecting aerosol constituents is also unknown; however, the drying step of the latter method might cause the loss of any volatile compounds and inadvertently prevent their presence in the ultimate fraction. The final collection method, “cotton filters,” was also cited only once (2%; 1/49). In essence, this method replicates the CFP method, although it provides a different and potentially less efficient sorbent (owing to varying pore sizes) than the glass fiber material of the CFP. Moreover, in this case, a water‐based protic solvent (PBS) was used to solubilize the captured ACM; thus, it is likely that the final fraction mainly contained water‐soluble constituents adhered to the cotton filters.

Aerosol collection methods (including solvents) were not the only parameter to vary among the 47 publications. There was also large heterogeneity among other key study elements, including the e‐cigarette devices, e‐liquids, smoking machines, puffs taken, and vaping regimens (as exemplified in Figures 3 and 4). Thus, while it is the prerogative of the institute undertaking the research to employ the materials and experimental conditions that meet its needs, it might be beneficial to promote the use of certain standards, such as topography research‐based vaping regimens, in order to standardize common aspects of aerosol generation, as was done for cigarettes in the past (CMR, 2018; Health Canada, 1999; ISO, 2012). Published recommendations from the Cooperation Centre for Scientific Research Relative to Tobacco (CORESTA) could also be leveraged in this regard (CORESTA, 2015).

More notably, however, as described above, the publications evaluated as part of this survey reported very limited chemical characterization data on the collected aerosols, if any at all. We assume that this type of analysis was generally not performed; however, it is theoretically possible that the data have just not been reported. Nevertheless, nicotine was quantified in several of the publications despite the use of different collection methods, indicating that at least this one prominent aerosol constituent was present in the various fractions produced (i.e., AQEs, AQ‐ and DMSO‐soluble ACM, and condensate). Interestingly, in addition to performing aerosol collection via the bubble‐through methodology (into cell culture medium) for assessing cytotoxic potential, one publication also reported analogous activities—but by using isopropanol as the solvent instead—in order to investigate in parallel the transfer of flavor chemicals from the e‐liquid to the captured aerosol (Omaiye, McWhirter, Luo, Pankow, & Talbot, 2019). Although the AQE applied to the cell cultures in this case was not the subject of characterization, the use of a potentially more effective trapping solvent (isopropanol rather than cell culture medium) still resulted in relatively poor collection (>50% transfer efficiency) of this chemical family subtype. These findings suggest that the AQEs generated via the bubble‐through methodology might not accurately represent the native aerosol, at least in terms of flavor content. Linked to these results is a broader point in relation to flavors. Given their volatile nature, no collection method might be truly optimal to capture all flavor chemicals, although further research (as described below) is required to inform this discussion.

In light of the findings of this survey, our fundamental concern relates to the apparent dearth of information on the overall chemical characteristics of the collected e‐cigarette aerosols. Without knowing the chemical composition of the fraction(s) evaluated, it is not possible to draw strong conclusions on the associated in vitro data. Thus, in order to enhance the utility of such data in this context, we recommend that research efforts be focused on chemically characterizing the fractions generated by each type of collection method in a comprehensive fashion. This work should endeavor to identify the collection method(s) and solvent(s) that produce the fraction(s) most representative of the native aerosol that is amenable to evaluation in submerged cell culture‐based assays. Furthermore, the chemical stability of the collected aerosol fractions should also be studied in order to ascertain their shelf life and optimal storage conditions, given that samples might be transported and stored before use. Establishing these conditions would pave the way for greater standardization of the entire e‐cigarette aerosol collection process for in vitro applications and, critically, raise the value of existing and future in vitro data on this topic. Interestingly, two recent publications described non‐targeted screening methodologies (based on gas chromatography with time‐of‐flight mass spectrometry) that were used to characterize the trapped emissions of tobacco products and e‐cigarettes (Knorr et al., 2019; Rawlinson, Martin, Frosina, & Wright, 2017). We envisage that similar approaches could be employed in the research proposed here in order to uncover in greater detail the chemical composition of the various aerosol fractions.

## 5 CONCLUDING REMARKS

In the present PubMed® survey, we identified seven types of methods that were used to collect the aerosol from e‐cigarettes for assessment in submerged cell culture‐based in vitro assays. The different collection methods (and associated solvent systems) have been appraised here to some extent by using the limited analytical data reported in the 47 publications themselves as well as supporting data generated elsewhere; however, we call for more comprehensive research into the chemical nature of the fractions produced in order to enhance our knowledge of their composition. In addition, we also endorse greater levels of standardization (in aerosol generation parameters, for example) in order to increase the levels of consistency among testing laboratories. Exploiting these opportunities would serve two purposes, both of which ultimately aim to support our comprehension of the effects of e‐cigarette‐derived aerosols on human health: (a) to improve our collective understanding of the in vitro assay data already reported in the public domain and (b) to ensure that the most effectual data are generated from in vitro studies in the future.
